# Have the tsunami and nuclear accident following the Great East Japan Earthquake affected the local distribution of hospital physicians?

**DOI:** 10.1371/journal.pone.0178020

**Published:** 2017-05-25

**Authors:** Saori Kashima, Kazuo Inoue, Masatoshi Matsumoto

**Affiliations:** 1 Department of Public Health and Health Policy, Graduate School of Biomedical and Health Sciences, Hiroshima University, Hiroshima, Japan; 2 Department of Community Medicine, Chiba Medical Center, Teikyo University School of Medicine, Chiba, Japan; 3 Department of Community-Based Medical System, Graduate School of Biomedical and Health Sciences, Hiroshima University, Hiroshima, Japan; University of Hyogo, JAPAN

## Abstract

**Objective:**

The Great East Japan Earthquake occurred on 11 March 2011 near the northeast coast of the main island, ‘Honshu’, of Japan. It wreaked enormous damage in two main ways: a giant tsunami and an accident at the Fukushima Daiichi Nuclear Power Plant (FDNPP). This disaster may have affected the distribution of physicians in the region. Here, we evaluate the effect of the disaster on the distribution of hospital physicians in the three most severely affected prefectures (Iwate, Miyagi, and Fukushima).

**Methods:**

We obtained individual information about physicians from the Physician Census in 2010 (pre-disaster) and 2012 (post-disaster). We examined geographical distributions of physicians in two ways: (1) municipality-based analysis for demographic evaluation; and (2) hospital-based analysis for geographic evaluation. In each analysis, we calculated the rate of change in physician distributions between pre- and post-disaster years at various distances from the tsunami-affected coast, and from the restricted area due to the FDNPP accident.

**Results:**

The change in all, hospital, and clinic physicians were 0.2%, 0.7%, and −0.7%, respectively. In the municipality-based analysis, after taking account of the decreased population, physician numbers only decreased within the restricted area. In the hospital-based analysis, hospital physician numbers did not decrease at any distance from the tsunami-affected coast. In contrast, there was a 3.3% and 2.3% decrease in hospital physicians 0–25 km and 25–50 km from the restricted area surrounding the FDNPP, respectively. Additionally, decreases were larger and increases were smaller in areas close to the FDNPP than in areas further away.

**Conclusions:**

Our results suggest that the tsunami did not affect the distribution of physicians in the affected regions. However, the FDNPP accident changed physician distribution in areas close to the power plant.

## Introduction

On 11 March 2011, an extremely strong earthquake, recorded as 9.0 on the Richter scale, occurred offshore of the northeast Pacific coast, ‘Sanriku’, of the main island, ‘Honshu’, of Japan. This earthquake, referred to as the Great East Japan Earthquake, triggered a giant tsunami, which was 9.3 meters at its highest and caused catastrophic damage to the Tohoku region in northeast Japan [1]. According to the report by the National Police Agency of Japan as of 9 December 2016, there had been 15,893 reported deaths in 12 prefectures and 2,556 missing persons in 6 prefectures attributed to the disaster [2]. Additionally, this disaster created one of the worst radiation leakage accidents, which was rated as the 7th (maximum) caution level on the International Nuclear Events Scale because of the high level of radioactive substances released from the Fukushima Daiichi Nuclear Power Plant (FDNPP) [3]. According to the report by the United Nations Scientific Committee on the Effects of Atomic Radiation (UNSCARE), iodine-131 and caesium-137, two major hazardous radionuclides, were released into the atmosphere in the ranges of 100 to 500 petabecquerels (PBq) and 6 to 20 PBq, respectively [4]. Such a massive and complex disaster inevitably has negative impacts on people’s health and daily life.

Long-term shortages of healthcare resources, as well as regional economic downturn or industry decline, are predictable following such events. Although physicians and medical staff will play an important role in reconstructing local health services in the area [5], it is likely that securing adequate numbers of healthcare professionals may be difficult after such a large disaster. Furthermore, many of the areas affected by the earthquake were medically underserved even before the disaster because they are rural regions [6, 7]. After the earthquake, according to the Japanese media, the situation was reportedly exacerbated, particularly in areas near the FDNPP, because of damage to infrastructure and radioactive contamination [8–10]. Indeed, one recent study reported changes in the geographic distribution of secondary care nursing staff following the Great East Japan Earthquake [11]. In addition, in our previous study, we evaluated physician’s characteristics susceptible to migration from the areas surrounding the nuclear power plant, and we found that less-aged physicians in the areas were more likely to decrease than other physicians [12]. However, we have not studied the overall flow of physicians before and after the disaster. Particularly there is a lack of knowledge about the physicians' migration pattern not only from the areas of nuclear accident, but also from the tsunami-affected areas.

Therefore, we evaluated the effects of the Great East Japan Earthquake on the distribution of physicians within the most severely affected prefectures (Iwate, Miyagi, and Fukushima). Two major factors in this disaster may potentially impact physician distribution: the tsunami and the FDNPP accident. We analyzed changes in the number of hospital physicians at varying distances from the tsunami-affected area and from the FDNPP. Furthermore, we conducted an analysis based on two geographical units: municipality-based for demographic evaluation and hospital-based for geographic evaluation.

## Materials and methods

### Study area

We selected three prefectures (Iwate, Miyagi, and Fukushima) that were severely damaged by the Great East Japan Earthquake. Within the study area, the number of people killed was 15,826 (99.6% of the total killed), the number of people missing was 2,552 (99.8% of the total missing), and the number of people injured was 4,541 (73.8% of the total injured) [2]. Miyagi Prefecture was the most severely affected (killed: 9,540; missing: 1,232; injured: 4,145), followed by Iwate (killed: 4,673; missing: 1,123; injured: 213), and Fukushima (killed: 1,613; missing 197; injured: 183). The number of casualties as a result of the tsunami was greater than 14,100 people (92.4% of deaths caused by the Great East Japan Earthquake), but no one was killed by direct effect of radiation. More than 80,000 people were forced to evacuate the region by an order from the government [1].

### Physician data

Data regarding physicians were obtained from the Survey of Physicians, Dentists and Pharmacists compiled by the Ministry of Health, Labour and Welfare [13, 14]. Addresses of healthcare facilities were obtained from the Static Survey of Medical Institutions [15]. In Japan, the Ministry conducts a complete census of physicians every 2 years. All licensed physicians must register for this census. Every 3 years, the Ministry conducts The Static Survey of Medical Institutions. By law, all clinics and hospitals in Japan must report their activities and resources in this survey. We obtained permission from the Ministry to use data from both censuses for research purposes.

Because the earthquake occurred in March 2011, we obtained individual information about physicians from the Physician Census in 2010 (pre-disaster) and 2012 (post-disaster). We also obtained information about individual hospitals and clinics from the Static Survey in 2008 (pre-disaster) and 2011 (post-disaster).

In the study area, there were 11,686 physicians at pre-disaster and 11,646 physicians at post-disaster. We recognized registered license holders as ‘physicians’ if their main job was recorded as practicing in hospitals (including university hospitals, hospital physician) or clinics (clinic physician). For demographic and geographic analysis, we analyzed only hospital physicians since such data were not available for clinics.

We excluded hospital physicians whose prefecture number, city code, type of work, or facility code or name data were missing or inconsistent in the record. To ensure comparability between data in the pre- and post-disaster years, we excluded hospital physicians whose hospital was relocated to another city or changed its function from a hospital to clinic after the disaster. In the post-disaster census, 18 hospital physicians were registered temporarily as practicing in a clinic. We treated these physicians as hospital physicians for the analysis.

To count the number of physicians and measure the distance from hospital or municipality to the disaster site, we linked the physician census and medical institution census using the code or name of each physician’s working facility.

### Distance from disaster site

We measured distances from the disaster site to the physicians’ workplaces based on municipality (municipality-based analysis) and hospital (hospital-based analysis). We adopted these two methods for several reasons. In the municipality-based analysis, although the exact distance of each hospital from the disaster site cannot be calculated, the ratios of hospital physicians-to-population in each municipality can be. In contrast, hospital-based analysis enables the exact distance to be obtained, while physicians-to-population ratios cannot be calculated.

**Fig 1:**
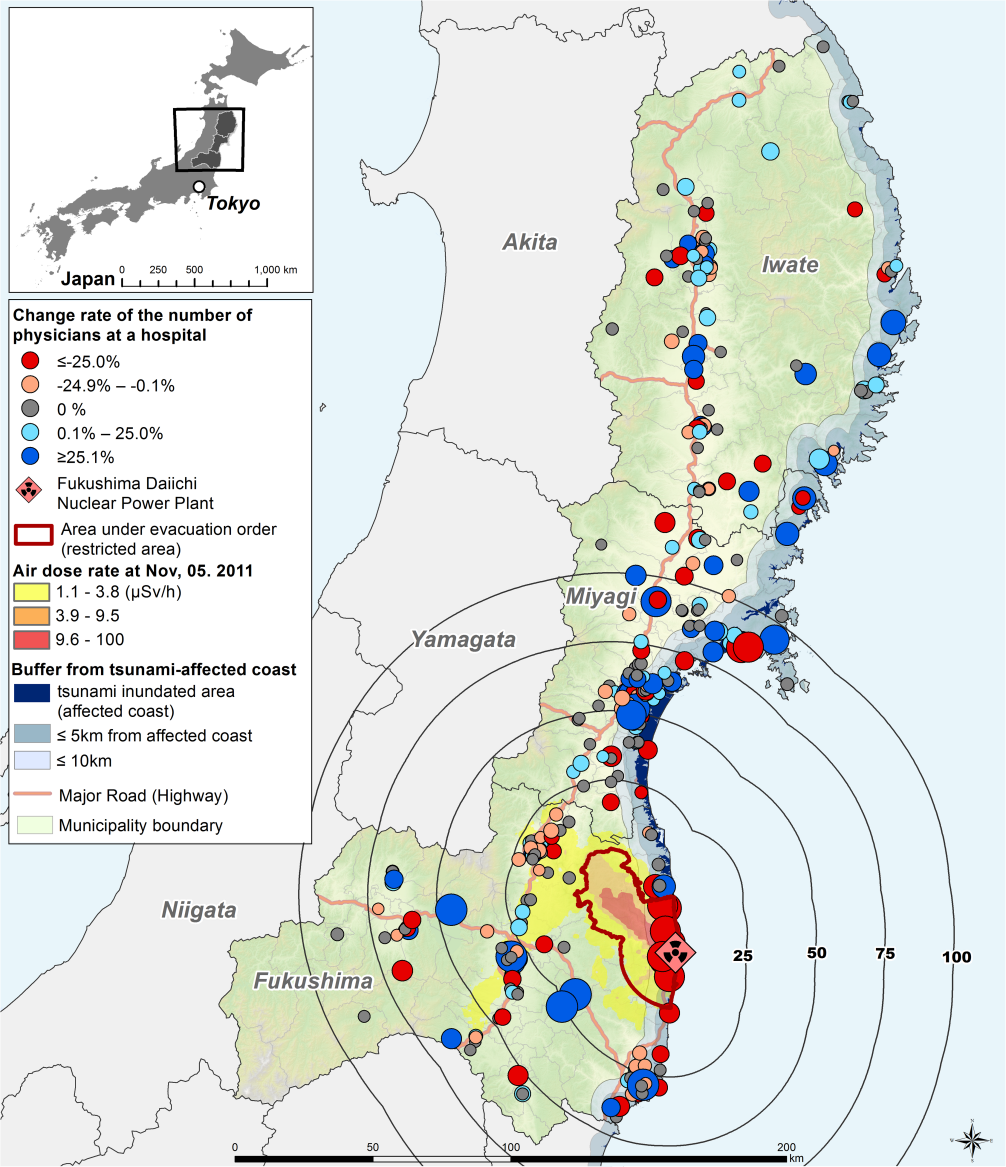
Map showing the geographical characteristics of disaster area and the change rate of physicians. The size of the symbol for change rate represents the magnitude of the change. Air dose rate was measured within 80 km of the nuclear power plant and data were acquired from the Japan Atomic Energy Agency.

In the analyses, we accounted for two types of disaster: the tsunami-affected coast and radiation leakage from the FDNPP. To precisely locate the tsunami-inundated area, 100 meter × 100 meter grid cell data (updated on 18^th^ April 2011) were obtained from the Geospatial Information Authority of Japan, Ministry of Land, Infrastructure, Transport and Tourism [16]. For the distance from the tsunami-affected coast (tsunami-distance), we adopted a borderline at the point where the tsunami reached furthest inland. Distance to each healthcare facility was classified into three categories: ≤5 km, 5–10 km, and >10 km. The area within 5 km included the inundated area. To measure the distance from the point of the FDNPP accident (FDNPP-distance), we used the distance from the radiation-polluted area because the straight distance to the power plant does not represent the extent of radioactive contamination because contamination levels depend on the wind direction. For the distance from the radiation-polluted area, we adopted a borderline of the area under evacuation order, which was designated by the government as of 22 April 2011 (Fig 1). The area under evacuation order, which is designated by the government as the ‘restricted area’, contained ‘restricted areas with penalties’ (the area within 20 km) and ‘deliberate evacuation areas’ (in which the residents can enter without penalties, but they are not permitted to live) [17]. The government ordered evacuation of the population from the radiation-polluted area based on air radiation dose rates, which were measured within 80 km of the FDNPP. The measurement was conducted as a part of the 4^th^ Airborne Monitoring Survey on 5 November 2011 by the Ministry of Education, Culture, Sports, Science and Technology, Japan [18]. The area under evacuation order remained a restricted area at the time of the physician census in December 2012. The distance from each healthcare facility to the restricted area due to the FDNPP accident was classified into five categories: 0 km (the zone including or within the area under evacuation order), 0–25 km, 25–50 km, 50–75 km, and 75–100 (inclusive)km. To account for both the tsunami and FDNPP accident simultaneously, we selected the areas within 5 km of the tsunami-affected coast and within 100 km of the restricted area surrounding the FDNPP, and conducted an analysis of these areas.

In the municipality-based analysis, we classified the distance of municipalities from the disaster site based on whether a whole or any part of the municipality was included within the distance specified. For instance, if a municipality included any areas ≤5 km in tsunami-distance and >0 and ≤ 25 km in FDNPP-distance, the municipality was placed in the category of ≤5 km in tsunami-distance and 0–25 km in FDNPP-distance. Administrative boundaries of municipalities for 2011 were obtained from the Ministry of Land, Infrastructure, Transport, and Tourism. In the hospital-based analysis, we used the address of each hospital to calculate the distance from the disaster site. The distance was measured using ArcGIS version 10.1 (ESRI Japan Inc.).

### Population data

Population data for each municipality were obtained from the Survey on the Basic Register of Residents, conducted by the Ministry of Internal Affairs and Communications, in 2009 (pre-disaster) and 2012 (post-disaster).

### Statistical analysis

Because of data availability, we focused on hospital physicians. First, to evaluate overall trends, we investigated the demographic characteristics and information regarding medical resources for the entire study area. Next, we conducted a municipality-level analysis, in which we calculated the rate of change from pre- to post-disaster year in the number of hospital physicians, classified according to the tsunami-distance, the FDNPP-distance, and the FDNPP-distance within tsunami-affected area. Finally, we conducted a hospital-based analysis, in which we calculated the rate of change in the number of physicians classified according to the tsunami-distance, the FDNPP-distance, and the FDNPP-distance within tsunami-affected area. In addition, to evaluate variability in rates of change in the ratio of hospital physicians to total population, we calculated the median and interquartile range for each hospital. Furthermore, we treated the distance from the disaster site as a continuous variable, evaluating the rate of change in physicians against the distance using scatter plots with a cubic polynomial regression line.

To remove the potential effects of the FDNPP accident from our evaluation of tsunami-distance, seven municipalities located within the restricted area due to the FDNPP accident were excluded from the municipality-based analysis using tsunami-distance. We also excluded physicians at these hospitals from the hospital-based analysis using tsunami-distance. In addition, since areas further from the FDNPP were less affected by the accident, the analysis in FDNPP-distance was only evaluated within 100 km from the restricted area.

For context, we recorded the number of clinics and clinic physicians for the entire study area. We obtained data on the number of clinic physicians from the Static Survey of Medical Institutions because we could not obtain information on the working places of clinic physicians in the pre-disaster year from the Physician Census.

Descriptive statistical analysis was conducted in IBM SPSS Statistics version 22 (IBM Japan Inc., Tokyo, Japan) and graphical analysis was conducted with KaleidaGraph version 4.5 (HULINKS Inc. Tokyo, Japan).

## Results

### Geography of the disaster area

Fig 1 shows the pre-disaster hospital locations, the geographical characteristics of the tsunami-affected coast, and the post-disaster air radiation dose rate (8 months later). As shown, hospitals were located both in coastal and inland areas, which were populous. The tsunami-affected area spread along the coastline, and reached near-coastal inland areas (at maximum, about 11 km along a river from the coast). The rates of change in physicians at each hospital are also shown in Fig 1. The magnitude of decrease, represented by the size of the symbols, was larger in Fukushima than in the other prefectures. In addition, the largest decrease was observed in the area with an air radiation dose rate of over 1.1 μSv/h.

### Total number of practicing physicians

Basic characteristics of the study area during pre- and post-disaster years are shown in Table 1. At post-disaster, the total population within the study area decreased by 2.0%. During this period, nine hospitals were closed or stopped practicing, seven of which were located within 20 km of the FDNPP, i.e., the restricted area. The total number of practicing physicians increased slightly (0.2%) during this period. Specifically, hospital physicians increased, whereas clinic physicians decreased. However, there was a trend for lower rates of change in the study area than nationally (shown in parentheses) amongst both hospital and clinic physicians. The physicians-to-population ratios tended to increase in both physician categories, although that of clinic physicians increased less than that of hospital physicians.

**Table 1:** Demographic characteristics and number of hospitals and physicians in pre- and post-disaster year.

	Pre-disaster, N	Post-disaster, N	Change rate, % (National change rate^§^)
Population	5,725,977	5,613,131	-2.0
Hospital	375	366	-2.4 (-0.7)
Clinic^†^	3,959	3,854	-2.7 (-0.3)
All physicians^‡^	11,008	11,026	0.2 (3.0)
at hospital	7,063	7,109	0.7 (4.1)
at clinic	3,945	3,917	-0.7 (1.1)
Physicians per 100,000 population^‡^	192	196	2.1
at hospital	123	127	3.3
at clinic	69	70	1.4

† The number of clinics was obtained from the Static Survey of Medical Institutions, and included a special elderly nursing home.

‡ The physicians at the nursing home were not counted in this number.

§ The parentheses indicate the national changes.

### Municipality-based analysis

Table 2 shows the municipality-based analysis of number of hospitals and hospital physicians, classified according to tsunami-distance, FDNPP-distance, and FDNPP-distances within the tsunami-affected area. There was a slight decrease in hospital physicians in the area closest to tsunami-affected coast (≤5 km), but increases in other areas. Hospital physicians-to-population ratios increased slightly in all three areas, including the closest area (≤5 km). In contrast, hospital physicians decreased in areas 0 km, 0–25 km, and 25–50 km from the restricted area surrounding the FDNPP. There was also a decrease in hospital physicians-to-population ratios in areas 0 km from the restricted area. There were dramatic reductions in the number of physicians and physicians-to-population ratios in areas affected by both the tsunami and FDNPP accident.

**Table 2:** Population sizes and number of hospital physicians, classified according to the distance from the tsunami-affected coast and the nuclear power plant in 131 municipalities (municipality-based analysis).

	Municipality, N	Hospital physicians, N			Physicians per 100,00 population, N		
		Pre- disaster	Post- disaster	Change rate (%)	Pre- disaster	Post- disaster	Change rate (%)
**Tsunami-distance (T) (municipality N = 124)** ^†^							
T: ≤5 km	33	1,800	1,793	-0.4	96	98	2.9
T: 5–10 km	13	1,753	1,842	5.1	190	196	2.8
T: >10 km	78	3,411	3,422	0.3	123	126	2.6
**Fukushima Daiichi Nuclear power plant (FDNPP) -distance (F) (municipality N = 92)**							
F: 0 km ^‡^	10	191	102	-46.6	117	68	-42.1
F: 0–25 km	18	1,930	1,874	-2.9	187	189	0.8
F: 25–50 km	23	1,446	1,414	-2.2	179	179	0.0
F: 50–75 km	24	4,050	4,169	2.9	252	256	1.6
F: 75–100 km	17	694	721	3.9	137	148	8.2
**FDNPP-distance within tsunami-affected area (municipality N = 25)**							
T: ≤5 km & F: 0 km^‡^	6	176	89	-49.4	130	71	-44.8
T: ≤5 km & F: 0–25 km	5	627	606	-3.3	150	152	1.2
T: ≤5 km & F: 25–50 km	3	237	242	2.1	156	161	3.1
T: ≤5 km & F: 50–75 km	8	1359	1375	1.2	189	190	0.3
T: ≤5 km & F: 75–100 km	3	326	317	-2.8	150	159	5.9

^†^ The seven municipalities located within the restricted area due to the nuclear accident were excluded from analysis.

^‡^ The area including the restricted area due to the nuclear accident. Information for this area is for reference, since it includes residential restriction areas and the restricted residence area.

### Hospital-based analysis

**Fig 2:**
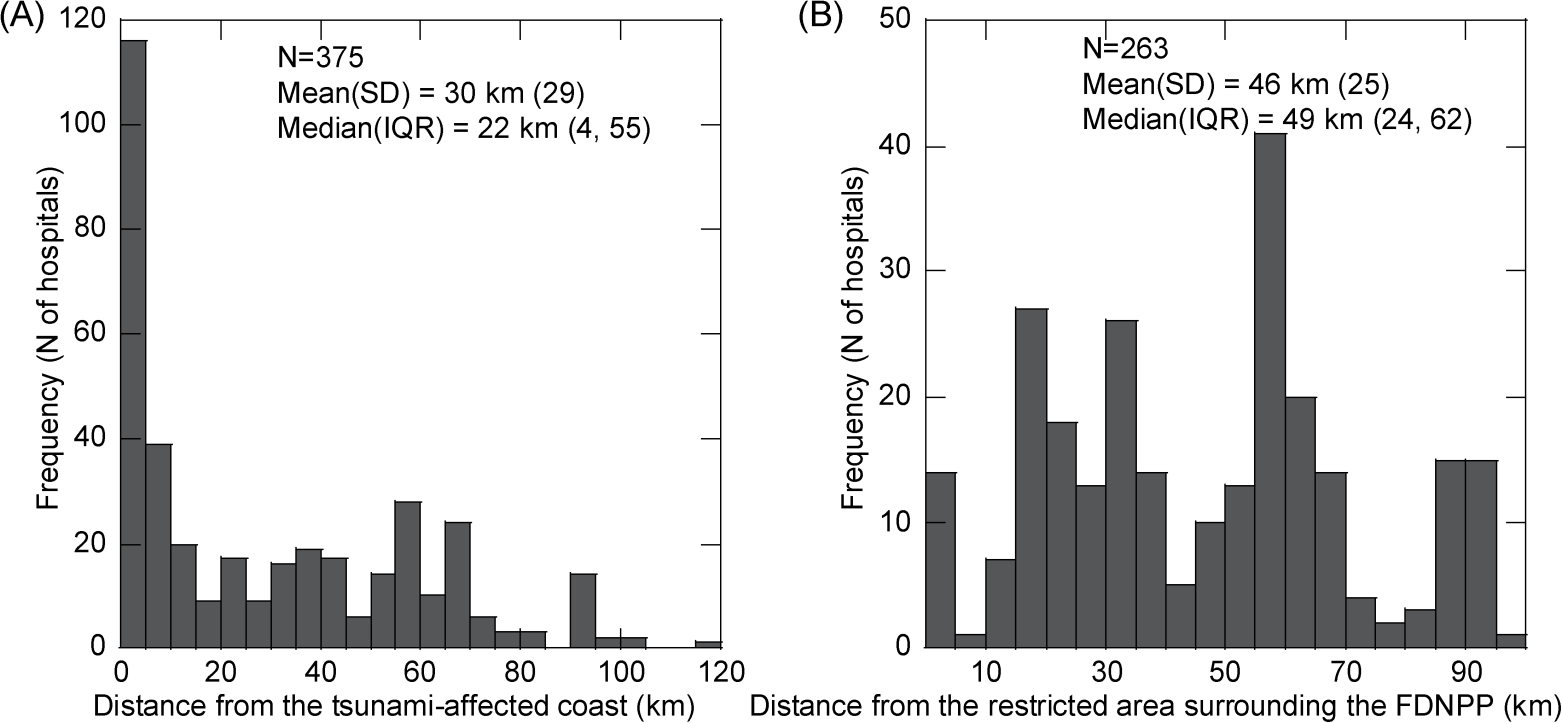
Distributions of distance from disaster site to each hospital in the pre-disaster year (hospital-based analysis). (A) Distance from the tsunami-affected coast and (B) from the restricted area surrounding Fukushima Daiichi Nuclear power plant (FDNPP). IQR, inter quartile range (25th percentile, 75th percentile); SD, standard deviation.

Fig 2 shows the distributions of distances of each hospital from the tsunami-affected coast (A) and the restricted area surrounding the FDNPP (B). As shown in Fig 2(A), within the studied range of 0–120 km from the tsunami-affected coast, almost 40% of the hospitals are located within 5 km (30.9%) and 5–10 km (10.4%). As shown in Fig 2(B), 7 hospitals (2.7%), 60 hospitals (22.8%), and 68 hospitals (25.9%) are located within 0 km, 0–25 km, and 25–50 km from the FDNPP, respectively.

Table 3 shows the hospital-based analysis of number of hospitals and hospital physicians, classified according to the tsunami-distance, FDNPP-distance and FDNPP-distance within tsunami-affected area. In this analysis, there was no change in the number of hospital physicians in the area within 5 km, while there were increases in areas within 5–10 km and >10 km of tsunami-distance. All seven hospitals closed or stopped practicing and all 45 hospital physicians left the designated restricted area. Hospital physicians decreased in areas ≤50 km and increased in areas over 50 km from the restricted area. The decrease in number was remarkable (-10.4%) at both the area affected both by the tsunami (≤5 km) and the FDNPP accident (0–25 km).

**Table 3:** Number of hospitals and physicians classified according to the distance from the tsunami-affected coast and the nuclear power plant in the pre- and post-disaster years (hospital-based analysis).

	Hospitals, N			Hospital physicians, N		
	Pre- disaster	Post- disaster	Change rate (%)	Pre- disaster	Post- disaster	Change rate (%)
**Tsunami-distance (T) (hospital N = 368)** ^**†**^						
T: ≤5km	109	107	-1.8	1,742	1,742	0.0
T: 5–10 km	39	39	0.0	1,491	1,544	3.6
T: >10 km	220	220	0.0	3,785	3,823	1.0
**Fukushima Daiichi Nuclear power plant (FDNPP)-distance (F) (hospital N = 263)**						
F: 0 km ^‡^	7	0	-100.0	45	0	-100.0
F: 0–25 km	60	60	0.0	1,154	1,116	-3.3
F: 25–50 km	68	68	0.0	1,009	986	-2.3
F: 50–75 km	92	92	0.0	2,709	2,778	2.5
F: 75–100 km	36	34	-5.6	419	434	3.6
**FDNPP-distance within tsunami-affected area (hospital N = 93)**						
T: ≤5 km & F: 0 km ^‡^	7	0	-100.0	45	0	-100.0
T: ≤5 km & F: 0–25 km	15	15	0.0	135	121	-10.4
T: ≤5 km & F: 25–50 km	24	24	0.0	232	231	-0.4
T: ≤5 km & F: 50–75 km	35	35	0.0	934	943	1.0
T: ≤5 km & F: 75–100 km	12	10	-16.7	199	193	-3.0

^**†**^ The seven hospitals located within the restricted area due to the nuclear accident were excluded from the analysis.

^‡^ The area including the restricted area due to the nuclear accident. Information for this area is for reference, since it includes residential restriction areas and the restricted residence area.

**Fig 3:**
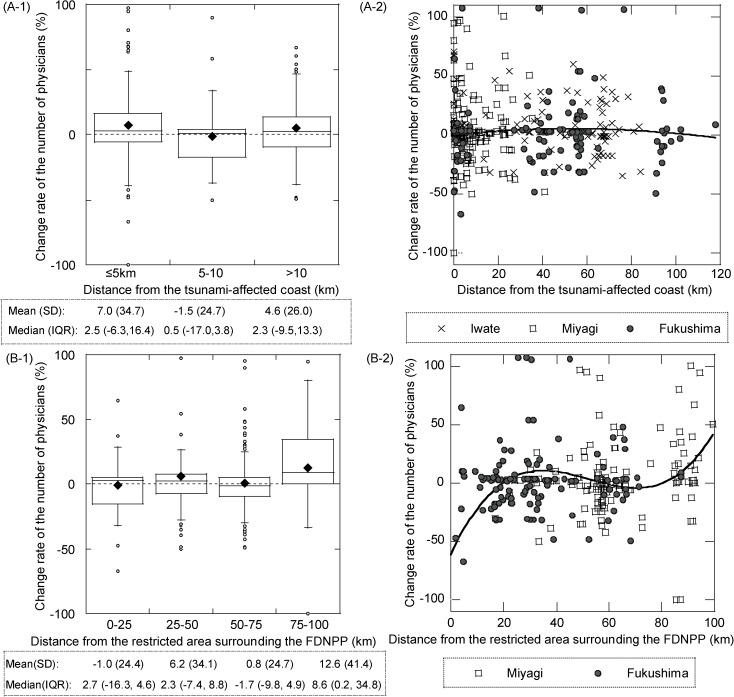
Boxplots and scatter plots showing hospital physicians-to-population ratios against distance from disaster site. (A) Distance from the tsunami-affected coast and (B) from the restricted area surrounding Fukushima Daiichi Nuclear power plant (FDNPP) (hospital-based analysis). Municipality-based population data were used, while physician data were hospital-based. Seven hospitals located within the restricted area were excluded from the analysis with tsunami-distances. In Fig A-1 and B-1, the whiskers represent the 1.5 * inter quartile range ± the 25th and 75th percentile. The black diamond represents the mean of all of the data. IQR, inter quartile range (25th percentile, 75th percentile); SD, standard deviation.

Fig 3 shows the variability among hospitals in the rate of change of physicians-to-population ratios. Tsunami-distance (A) and FDNPP-distance (B) were treated as categorical variables in the boxplots (1) and as continuous variables in the scatter plots (2). The interquartile range of the rate of change did not change significantly with increased distance from the tsunami-affected coast (Fig 3A-1). When we treated the tsunami-distance as a continuous variable (Fig 3A-2), the cubic polynomial regression line was almost linear. Although variability in the rate of change was larger in closer areas than in more distant areas, variability was not significantly dependent on tsunami-distance. The interquartile range of the rate of change moved up slightly with increased FDNPP-distance (Fig 3B-1). When we treated the FDNPP-distance as a continuous variable (Fig 3B-2), the rate of increase was lower in the closest areas than other areas. Regardless, we excluded physicians at seven hospitals within the designated restricted area, following which the rate of increase was lowest in Fukushima (Fig 3B-2, gray circles) than in Miyagi Prefecture.

## Discussion

In this study, we evaluated changing distributions of physicians in the aftermath of the Great East Japan Earthquake. We analyzed the distributions at varying distances from the tsunami-affected coast and the restricted area due to the FDNPP accident. The number of physicians did not decrease in any subgroup of tsunami-distance, and actually increased when we adjusted for changes in the population. In contrast, the number of hospital physicians decreased within 50 km in FDNPP-distance. The decrease was most remarkable in areas 0–25 km away, except the area within the designated restricted area. In addition, a wider distribution of rates of change was observed in the area within 50 km of FDNPP-distance compared with more distant areas.

These results suggest the tsunami and nuclear leakage accident affected local areas differently. While a marked decrease was observed within the area affected by both the tsunami and FDNPP accident, the FDNPP potentially had a greater effect on the number of physicians than did the tsunami because the number of physicians was stable in tsunami-affected areas, excluding the influence of the FDNPP accident. Although the tsunami, a direct natural disaster, caused severe damage in coastal areas, communities were able to embark on reconstruction soon after the tsunami. In contrast, nuclear accidents, an artificial and secondary disaster, scatter radioactivity that may last years into surrounding areas, keeping people away from the area. Therefore, although both disasters originated from the same earthquake, the nuclear accident causes more serious and chronic influence in surrounding communities than the tsunami. Few studies have evaluated these differences.

In this study, we evaluated physician migration patterns in the areas affected by the tsunami and nuclear accident. The earthquake itself has also damaged the area, although the extent of the damage was far less than that of the tsunami. For example, 92.4% of deaths by the Great East Japan Earthquake were caused by drowning, 4.4% was by crush injuries, 1.1% was by burns, and 2.0% was by unspecified causes [1]. Thus, the effects of the earthquake itself on the number of physicians are potentially small in this disaster situation.

It is noteworthy that the number of hospital physicians did not change in the area within 5 km of the tsunami-affected coast, even though two hospitals were closed or stopped practicing between the pre- and post-disaster years. In addition, the number of hospital physicians increased in areas 5–10 km from the tsunami-affected coast. The increased number is potentially influenced by temporarily placed physicians, who were dispatched to the affected area, particularly the tsunami-affected area, immediately after the disaster. According to official reports, 12,385 health professionals (2,720 teams) were dispatched to the area during the year after the disaster [19]. However, most of such temporarily placed physicians returned to their original facilities within several months. In addition, it was reported that the main activity of the dispatched medical teams terminated 4 months after the earthquake [20]. Thus, the effect of such temporary physicians on present findings would be minimal by the end of 2012 when we obtained our post-disaster data.

On the other hand, decreased numbers of physicians were observed in the areas closest to the FDNPP. In the pre-disaster year, 45 physicians were working in seven hospitals within 20 km of the FDNPP, an area which was later designated as a restricted area by the government. Thus, they must have moved to areas further than 20 km from the FDNPP. However, a decrease in physicians was also observed in the areas 0–25 km away from the restricted area. These findings are consistent with a previous study that evaluated trends in nurse distributions after the same disaster [11]. The authors reported a decrease in nurses in the areas most affected by radiation. Fear of radiation might be even greater in physicians than the general population, discouraging them from practicing in the area.

There are a few studies that have reported changes in the number of physicians after similar catastrophic disasters. Following hurricane Katrina in the United States, total bed capacity decreased by about 80% approximately six months after the disaster [21]. Even after one year, the number of physicians in the affected parishes in New Orleans was 48% that of pre-disaster levels [22]. Previous studies suggest that economic concerns appeared to be a key factor in the failure of physicians to return to the Katrina-affected areas [22, 23]. Although it may be differ between the situation of hurricane Katrina and the Great East Japan Earthquake, it is noteworthy that the number of physicians did not change in the tsunami-affected area after the earthquake. One possible explanation may be due to universal health insurance of Japan. In Japan, therefore, people may visit clinics and hospitals with being covered by a certain insurance, and clinics and hospitals may get fair reimbursement fee even at disaster areas [24]. In addition, the Japanese government exempted disaster victims from paying monthly premiums and out-of-pocket payments for medical care [6, 25]. Furthermore, the government provided financial support for medical institutions to restore damaged buildings, in accordance with the Special Reconstruction District Act [6, 25]. This financial support might have potentially contributed to retaining physicians in affected areas.

We adopted two analysis methods to examine the influence of this earthquake. One was municipality-based and the other was hospital-based, both of which have their own advantages and disadvantages. The former enabled calculation of physician-to-population ratios, and the latter provided precise geographic analysis. The trends in physician distributions were, by and large, similar in the two methods. We used the detailed addresses of the hospitals where physicians were working. We measured the detailed distribution by calculating the means, medians, and interquartile ranges for each hospital, according to their distance from the tsunami-affected coast and the restricted area due to the FDNPP accident (Fig 3). We observed that the rate of change in the number of physicians before and after the disaster varied according to geographic FDNPP-distance. It is noteworthy that the rate of change was different among hospitals located at a similar FDNPP-distance. The standard deviations of the change rates at hospitals close to the restricted area surrounding FDNPP were larger than those further away (Fig 3B). This suggests that there was more variation in mean changes of physicians among closer hospitals than among more distant hospitals. Variability might be related to hospital characteristics, such as size and whether it is public or private. Further study is required that takes hospital characteristics into account to assess and avoid bias in the results.

All prefectures studied had already faced a shortage of physicians, even before the disaster, because of their rural location [6, 7]. The numbers of physicians per 100,000 people in 2010 (pre-disaster) were 193.7 in Iwate, 222.0 in Miyagi, and 191.2 in Fukushima. These were all under the national average (230.4). The pattern of changes in physician distributions differed among the prefectures. In Iwate, a decrease in physicians was observed regardless of the tsunami or FDNPP accident (Figs 1 and 3). In contrast, in Fukushima the decrease was particularly prominent in areas close to the nuclear power plant. Although the number of physicians increased in the surrounding area, hospitals located within 50 km of the restricted area surrounding the FDNPP, especially where the air dose rate was 1.1μSv/h, experienced decreases of more than 25% (Fig 1).

In this study, we obtained population data from a survey on the basic register of residents for municipality-level analysis. Some residents have not changed their registration address even though they have moved to other cities after the disaster. This might be particularly true for areas within the restricted area surrounding the FDNPP. As such, the population size in the post-disaster year may be overestimated. This would mean that the rate of change in the number of physicians per unit population is smaller than estimated. However, we used the population data at municipality level, so if residents moved within the same municipality, bias would be minimal.

There are other limitations to this study. We were only able to obtain population data at municipality level. We could not obtain data in smaller geographic units, such as small census blocks. In the hospital-based analysis, because we used the population of the municipality in which the hospital was located, we could not directly calculate the number of hospital physicians per population in the hospital catchment area. Despite of these limitations, our findings are novel and tell how the two great disasters affect the distribution of human resource for health following the Great East Japan Earthquake. Our data would be a reference for other countries which are preparing for such disasters.

## Conclusions

The differences in the distributions of the physicians before and after the tsunami were not substantial. Supportive action from other prefectures may have contributed to the lack of physicians in these areas. However, the number of hospital physicians in areas close to the restricted area surrounding the nuclear power plant decreased significantly, even after adjusting for population decreases. These results suggest a substantial difference in seriousness of the consequences of the two main disasters following the earthquake. Continuous observation is required in the affected areas, particularly near the nuclear power plant that caused radiation leakage.
